# Predicting the duration of reach-to-grasp movements to objects with asymmetric contact surfaces

**DOI:** 10.1371/journal.pone.0193185

**Published:** 2018-02-22

**Authors:** Rachel O. Coats, Raymond J. Holt, Geoffrey P. Bingham, Mark A. Mon-Williams

**Affiliations:** 1 School of Psychology, Faculty of Medicine and Health, University of Leeds, Leeds, United Kingdom; 2 School of Mechanical Engineering, Faculty of Engineering, University of Leeds, Leeds, United Kingdom; 3 Department of Psychological and Brain Sciences, Indiana University, Bloomington, Indiana, United States of America; University of Exeter, UNITED KINGDOM

## Abstract

The duration of reach-to-grasp movements is influenced by the size of the contact surfaces, such that grasping objects with smaller contact surface areas takes longer. But what is the influence of asymmetric contact surfaces? In Experiment 1a, participants reached-to-lift wooden blocks off a table top, with the contact locations for the thumb and index finger varying in surface size. The time taken to lift the block was driven primarily by the thumb contact surface, which showed a larger effect size for the dependent variable of movement duration than the index finger’s contact surface. In Experiment 1b participants reached-to-grasp (but not lift) the blocks. The same effect was found with duration being largely driven by contact surface size for the thumb. Experiment 2 tested whether this finding generalised to movements towards conical frusta grasped in a different plane mounted off the table top. Experiment 2 showed that movement duration again was dictated primarily by the size of the thumb’s contact surface. The thumb contact surface was the visible surface in experiments 1 and 2 so Experiment 3 explored grasping when the index finger’s contact surface was visible (participants grasped the frusta with the index finger at the top). An interaction between thumb and finger surface size was now found to determine movement duration. These findings provide the first empirical report of the impact of asymmetric contact surfaces on prehension, and may have implications for scientists who wish to model reach-to-grasp behaviours.

## Introduction

A majority of neurologically intact adult humans possess the ability to reach-and-grasp objects in a highly precise manner. The skills inherent within this behaviour can be seen in the stereotypical movements of the digits as they approach an object, before the fingertips exert the forces required to interact with the item (the forces being a function of the movement goal). The stereotypical nature of the movements can be exploited by the scientist to make probabilistic predictions about the behaviour that will emerge as a function of the individual and the task. Thus, a major goal within sensorimotor research is the identification of the factors within a task that alter prehensile behaviour in a reliable manner, and thereby allow for an accurate prediction of how an individual’s behaviour will alter as a function of changing environmental constraints.

One topic that has received great attention within the sensorimotor control literature concerns the relationship between task parameters and the duration of movement. Fitts first formalised the predictable relationship that exists between movement duration and the task parameters of target amplitude and size (with constants reflecting individual and task differences)- whereby increasing object distance results in movements of longer duration whilst increasing target size can reduce movement time [1]. The relationship between movement time, target distance and size has been well documented in aiming movements (moving a working point–often the tip of the index finger—from a starting position to a target location). Participants typically reach higher peak velocities when moving to further targets, but do not increase speed sufficiently to prevent a longer duration [2]. In aiming movements, decreasing the size of the target location potentially requires participants to implement error correction, and this situation appears to be the mechanism through which longer movement duration results.

There has been less investigation of the relationship between task constraints and movement duration with regard to reach-to-grasp (prehension) movements. Nevertheless, existing prehension research shows a similar pattern to aiming studies, such that participants reach higher peak velocities in movements to further objects but take longer to reach-and-grasp these more distant items. It has also been shown that decreasing the size of the surface available for digit placement in prehension causes an increase in movement time [3–4], as does decreasing the diameter of a cylindrical object [5]. It seems likely that this is driven by a mechanism similar to that operating in aiming movements–viz., increasing the accuracy demands raises the potential need for online corrections which causes longer duration movements. However, prehension has greater complexity than aiming. Mon-Williams and Bingham [3] have argued that reach-to-grasp movements necessarily entail both digit placement and object avoidance–a successful grasp generally requires the digits to avoid contact with the object during the approach phase (as collision can result in objects being knocked over). One consequence of collision avoidance is that larger objects can place higher demands on prehension (as there is more of the object to avoid; a problem because the hand has a limiting maximum aperture to which it can open). This suggests that increasing object size whilst keeping a constant contact surface size should result in longer movement duration, and indeed this has been shown [3]—though it is not an inevitable feature of prehension [6].

A detailed examination of these issues was recently reported by McIntosh, Mon-Williams and Tresilian [7]. In a series of experiments, McIntosh et al manipulated the depth (in the plane of the reach), height (orthogonal to the reach), and width (the grasped dimension) of objects in a reach-to-grasp task. The results showed lawful relationships that were consistent at the individual and group levels with increased duration when the objects were further and when the contact surface was smaller in either depth or height. The results also showed that duration increased for wider objects but only beyond a critical width that varied between individuals. The results were well captured by a two factor model where contact surface size and reach distance had discrete influences on movement duration. McIntosh et al provide a comprehensive review of relevant studies on this topic and suggest empirical and theoretical reasons for the two factor model.

Thus, there is good evidence to suggest that the duration of reach-to-grasp movements is some function of object distance, overall object magnitude and contact surface size. One issue that has not been addressed is the relationship between movement duration and contact surface size when the surface size is different for the thumb and index finger (when grasping objects with a pincer grip). This issue is of empirical interest for the scientist who wishes to predict movement time under a wide range of task constraints. Movement time might be a function of the size of the contact surface for the thumb, for the index finger, an average of the two surface sizes or a function of the smallest surface.

We wished to explore the prehensile behaviour elicited with objects that had asymmetric contact surfaces. There are a number of ways of conceptualising the control of prehension cf[8–11] but we approached these experiments from the perspective elucidated by Mon-Williams and Bingham [3]. Mon-Williams and Bingham suggested that prehension can be conceptualised as controlling the position, magnitude and orientation of an ‘opposition vector’ formed between the finger and thumb endpoints with the goal of alignment with the target object. The vector account assumes that the thumb is normally the default origin of the vector as it has evolved in humans to provide the large and stable base required for precision grips [12], and is most often the digit moving to a visible surface. Wing, Haggard & Flanagan suggested that positional control of the hand is aided by the thumb acting as a visual marker [10]. A number of studies have provided support for the idea that prehension involves visual guidance of the thumb [13–15] although others find evidence for visual guidance of the finger under certain conditions [16–18].

In the vector account of Mon-Williams and Bingham [3], prehension is envisaged as a complex ‘higher order’ behaviour that results from the merger of three ‘lower order’ actions (transport, rotation and grip formation) [3]. From this viewpoint, there are a myriad of different ways that the lower order actions can be temporally arranged. This conception paints a picture of prehension as a class of movements rather than a single behaviour. It also suggests that the organisation of prehension has much greater flexibility (because of its complex nature) than perhaps suggested by traditional accounts. For example, Mon-Williams and Bingham [3] showed that participants reaching-to-lift an object will sometimes stop the hand (and secure the object) before commencing lifting, but show ‘fly-through’ behaviour on other trials. The probability of observing ‘stop’ or fly-through’ actions is a function of the task constraints–with higher requirements for precision decreasing the probability of ‘fly-through’ reaches. This flexibility of hand aperture pattern is not well emphasised within the prehension literature but can be seen when different tasks are used in studies of reach-to-grasp behaviour [19].

We present data from four manipulations conducted across three experiments to determine the impact of contact surface asymmetry on prehensile behaviour (where participants reached to objects with asymmetric flat contact surfaces). A body of evidence has shown that synchronous movements of two aiming trajectories share a common duration dictated by the maximum accuracy constraint [20–22], and we were interested in exploring whether reach-to-grasp movements would show the same pattern. We were also interested in studying how the objects with asymmetric contact surfaces affected maximum grip aperture (as faster movements increase the risk of collision–a risk that can be mitigated by increasing the maximum aperture). We kept the width of the objects constant as it has been shown previously that increasing the width increases the collision hazard and causes changes in movement duration [7]. In Experiment 1a, we investigated reach-to-lift behaviours where we expected to see both ‘stop’ and ‘fly-through’ trials. In Experiments 1b, 2 and 3, we investigated reach-to-grasp behaviours where the hand stopped moving once the object was secured in the grasp (this allowed us to explore the pre-contact duration of the separate thumb and finger movements).

## Experiment 1a

### Method

#### Participants

Eight unpaid participants (undergraduate students) were recruited for the study (2 male, 6 female). Participants were aged between 20 and 25 years of age (mean = 22.5 years). Six participants were right handed and two were left handed. All had normal or corrected to normal vision, and none had any history of neurological deficit. Participants provided their written informed consent prior to their inclusion in the study. The study was approved by the School of Psychology, University of Aberdeen ethics committee, and was performed in accordance with the ethical standards laid down in the Declaration of Helsinki.

#### Experimental setup and procedure

**Fig 1:**
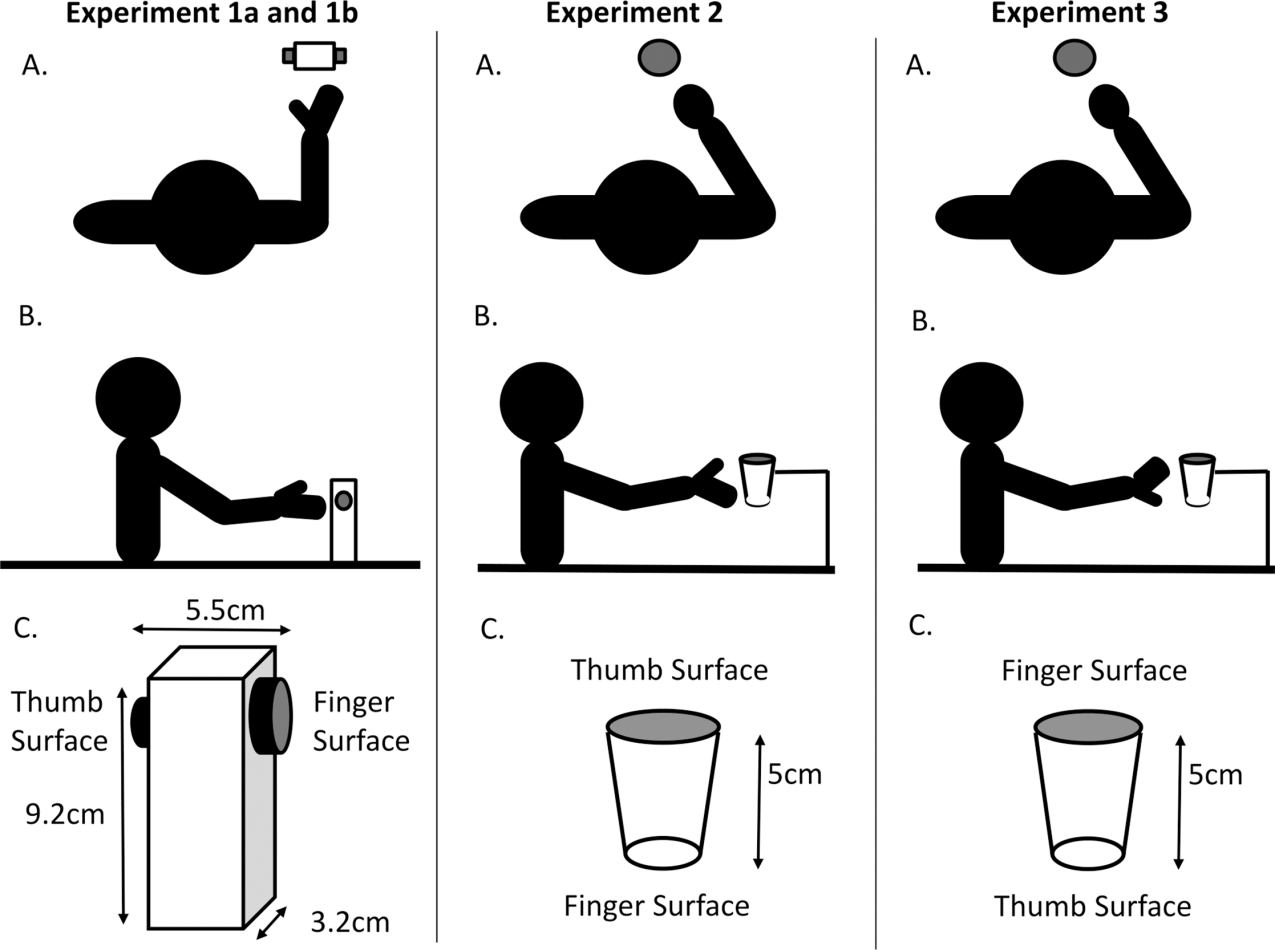
Experimental layout and target objects. Bird’s eye view (top), side view (middle) and schematic of the target objects (bottom) for Experiment 1a and 1b (left) Experiment 2 (middle) and Experiment 3 (right). In experiments 1a and 1b the object was grasped with the finger and thumb on either side. In experiment 2 the object was grasped with the thumb on top and finger underneath whist the opposite was the case for Experiment 3. In all cases the starting position was defined by a small (pea-sized) moulded grip on the table top along a sagittal plane (where the plane included the acromion process of the shoulder and the long axis of the object). In Experiment 1a, the target object was placed at one of three horizontal distances from the start point (10cm, 30cm or 50cm). In Experiment 1b it was placed 30cm from the start point. In Experiments 2 and 3 it was placed at one of three horizontal distances from the start point (10cm, 30cm or 50cm) but this time was 15cm above the table top.

The experimental task required participants to sit at a table and reach-to-lift (between their index finger and thumb) a cuboid wooden object with its long axis oriented vertically along a sagittal plane encompassing the acromion process shoulder of the participant’s preferred arm, such that the object was comfortable to reach with the preferred hand through simple extension of the arm. Wooden blocks were used (see Fig 1) as target objects. The objects were of the same dimensions (height 9.2cm x width 5.5 cm x depth 3.2 cm and all weighed <300g), but the size of the grasping surface to be contacted by the thumb and index finger was determined by thin sections of circular dowel (diameter of 3, 2, or 1 cm) attached to the side of the objects along the coronal plane. The middle of the dowel was set back by 1.5 cm from the top of the object. Different combinations of dowel were used so that each combination of contact surface size was possible (nine different objects in total).

The starting position was defined by a small (pea-sized) moulded grip on the table top 10cm from the table edge closest to the participant along a sagittal plane (where the plane included the acromion process of the shoulder and the long axis of the object). The grip was held by the participant between the thumb and index finger. The rest of the digits were folded into the palm, and the hand and forearm rested on the table top in a comfortable neutral posture. The lower arm was located approximately in line with the position of the block and reach-to-lift movements were performed with the preferred hand. This meant that the objects could be reached by straight extension of the arm in front of the participant and this resulted in the contact surfaces being the same distance from the starting position of the hand (i.e. the movements of the thumb and index finger were symmetrical). This arrangement resulted in the thumb surface being visible but not the index finger surface (so it was not possible to see both contact surfaces—in common with many grasping tasks). Nevertheless, although the participant could not see the finger surface they still knew the size of it as they could see the edge of what is essentially a predictable shape (i.e. they could infer the contact surface size from the edge that was visible). In this respect, participants ‘knew’ what size the contact surface was but couldn’t benefit from the feedback gained from seeing it directly, and more importantly from seeing their digit in relation to the surface. The three different sizes of dowel (3, 2, or 1 cm diameter) were contacted by the thumb, and by the index finger: therefore reaches were made to nine different thumb/finger contact-size combinations. The object to be lifted was placed at one of three horizontal distances from the starting point (10cm, 30cm or 50cm). Each participant performed 10 trials at each distance and contact surface size combination, resulting in a total of 270 trials. Trials were presented in blocks so that participants made 10 successive reaches to each randomly ordered trial configuration. At the beginning of each trial the experimenter counted “two, one, go” and the participant’s task was to reach to grasp the object at the ‘go’ signal and lift it off the table, as “quickly and accurately as possible”.

Data acquisition was initiated approximately one second before the experimenter’s verbal start command. Reach responses were recorded using four infra-red emitting diodes (IREDs) attached to the distal metacarpal joint of the index finger, the posterior aspect just distal to the distal metacarpal joint of the thumb, the wrist (styloid process of the radius), and to the wooden block participants were required to lift. The positions of the IREDs were recorded using an Optotrak movement recording system. Reach movements were recorded at 100Hz for 2 seconds. The stored data files were then filtered using a dual-pass Butterworth second order filter with a cut-off frequency of 16Hz (equivalent to a fourth order zero phase lag filter of 10Hz). The distance between the thumb and index finger IREDs was then computed (the aperture), allowing us to calculate the maximum grip aperture. The resultant tangential speed of the wrist, finger and thumb IREDs was calculated and movement onset was set at the point when the speed of the wrist exceeded 5cm/s. The resultant tangential speed of the IRED on the block was also calculated, with the onset of block movement determined as the point when the speed of the block exceeded 5cm/s. We defined the total movement time as the temporal difference between wrist onset and block onset.

#### Design

The experiment was a within-subjects design. All participants performed reaches to each combination of contact surface size at each distance. The order of size/distance combinations was randomised across and within the participants; however, participants performed 10 trials in succession at each configuration. The independent variables manipulated were the distance of the target and the contact surface size to be contacted by the finger and thumb. The dependent variables of interest were maximum grip aperture (MGA), and Movement Time (MT).

For each participant, the median value of the ten trials performed at each contact surface size and distance configuration was obtained and analysed for each dependent variable. Data were then entered into a repeated measures ANOVA, using thumb (3 contact surface sizes: 1, 2 and 3 cm), finger (3 contact surface sizes: 1, 2, and 3 cm) and distance (10, 30 and 50 cm) as within-subjects variables. Greenhouse-Geisser corrections were not required. Significant interactions are reported but in the interests of brevity no other interactions are described as they all failed to reach statistical significance. Means were calculated from the median values and the data not presented in Figures across the experiments can be found in Table 1.

**Table 1:** Means (and SDs) collapsed across distance for maximum grip aperture (MGA, mm), for all experiments.

		Thumb contact surface size		
	Finger contact surface size	Small	Medium	Large
**Experiment 1a**	**Small**	85 (7)	87 (6)	89 (6)
	**Medium**	88 (7)	88 (7)	90 (7)
	**Large**	88 (7)	90 (7)	90 (7)
**Experiment 1b**	**Small**	95 (13)	98 (10)	100 (9)
	**Medium**	99 (9)	102 (12)	105 (12)
	**Large**	99 (8)	105 (11)	104 (11)
**Experiment 2**	**Small**	89 (8)	92 (9)	94 (7)
	**Medium**	92 (8)	95 (8)	95 (8)
	**Large**	94 (9)	95 (6)	99 (8)
**Experiment 3**	**Small**	89 (6)	91 (7)	93 (7)
	**Medium**	90 (6)	93 (6)	93 (6)
	**Large**	92 (10)	94 (7)	96 (8)

### Results

#### Maximum grip aperture (MGA)

MGA was influenced by the contact surface for the thumb (F(2,14) = 16.30, p<0.001, $\eta_{p}^{2}$ = 0.7) and index finger (F(2,14) = 5.07, p = 0.02, $\eta_{p}^{2}$ = 0.42). MGA increased as the size of the thumb and index finger contact surface area increased. MGA was also significantly affected by distance (F(2,14) = 4.2, p = 0.04, $\eta_{p}^{2}$ = 0.38) with pairwise comparisons (with Bonferroni corrections) showing this was driven by larger MGAs in reaches to the middle distance compared to the far one.

#### Total movement time

**Fig 2:**
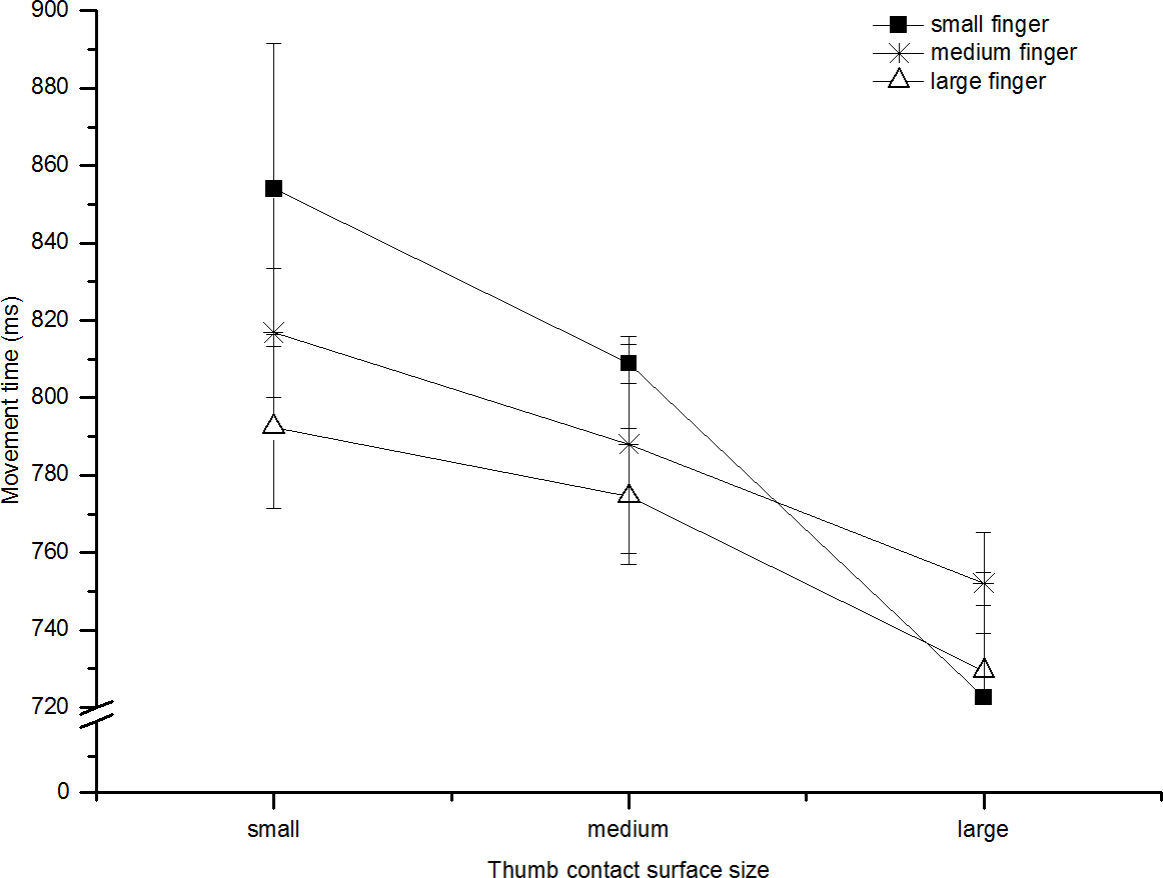
Movement time: Experiment 1a. Shows the average movement time (ms), for all participants in reaches to all contact surface sizes (collapsed across distance). The lines represent the finger contact surface (square = small, star = medium, triangle = large). Error bars represent standard error of the mean; these are calculated for within-subjects designs (Cousineau [23], with correction by Morey [24]).

Movement time (defined as wrist movement onset to block movement onset) increased as the amplitude of the movement increased (F(2,14) = 291.86, p<0.001, $\eta_{p}^{2}$ = 0.98). MT was unaffected by the contact surface for the finger (F(2,14) = 0.93, p = 0.42, $\eta_{p}^{2}$ = 0.12). However, there was an effect of contact surface for the thumb (F(2,14) = 9.46, p<0.001, $\eta_{p}^{2}$ = 0.58 see Fig 2) with total MT increasing as the contact size of the thumb decreased (consistent with the notion that more accuracy, and hence more time, is required to grasp a smaller surface).

## Experiment 1b

In Experiment 1b we studied whether the findings of Experiment 1a would generalise to a slightly different behaviour where participants reached-and-grasped the blocks but did not lift them off the tabletop. The use of a reach-to-grasp action meant that there were no ‘fly through’ actions so this also allowed us to investigate the duration of the thumb and index finger as we could identify the point at which they completed their approach to the target using velocity thresholds (not possible when there are fly through movements).

### Method

#### Participants

Eight unpaid participants (undergraduate and postgraduate students) were recruited for the study (3 male, 5 female). Participants were aged between 20 and 31 years of age (mean = 24.8 years). All participants were right handed. All had normal or corrected to normal vision, and none had any history of neurological deficit. Participants provided their written informed consent prior to their inclusion in the study. The study was approved by the School of Psychology, University of Leeds ethical review committee, and was performed in accordance with the ethical standards laid down in the Declaration of Helsinki.

#### Experimental setup and procedure

The experimental task was the same as that in Experiment 1a apart from the fact that participants were required to reach-to-grasp (remaining in contact with the contact surfaces of the target object until the trial had ended) rather than reach-and-lift the same cuboid wooden objects placed on the table in the same orientation. The only other difference was that we used the 30cm distance from the starting point only. Each participant performed 10 trials to each object, resulting in a total of 90 trials. Markers were placed as in Experiment 1a (although no marker was placed on the target object as it was not to be moved) and data were collected and filtered in the same way with the same parameters used for analysis.

Movement time of each IRED marker (finger, thumb and wrist) was calculated as the difference between movement onset (>5cm/s) and movement offset (<5cm/s). Due to the fact participants were asked to produce a qualitatively different movement (reach-to-grasp as opposed to reach-to-lift) and we did not use object movement to denote movement end. We now defined total movement time for each trial as the time when the finger, thumb and wrist had all come to a stop (the largest of the MT values for the three IREDs). We additionally studied the duration of the thumb and index finger as discussed previously.

#### Design

The experiment was a within-subjects design. All participants performed reaches to all nine target objects but target object order was randomised across the participants. The independent variables manipulated were the contact surface size to be contacted by the finger and thumb. The dependent variables of interest were maximum grip aperture (MGA), total movement time, and movement time of the thumb and finger. The median value of the ten trials performed to each target object were then entered into a repeated measures ANOVA, using thumb (3 contact surface sizes: 1, 2 and 3 cm), and finger (3 contact surface sizes: 1, 2, and 3 cm) as within-subjects variables. Means were again calculated from median values and all means not presented in Figures can be found in Table 1.

### Results

#### Maximum grip aperture (MGA)

MGA was influenced by the contact surface for the thumb (F(2,14) = 9.05, p<0.01, $\eta_{p}^{2}$ = 0.56) and index finger (F(2,14) = 5.04, p<0.001, $\eta_{p}^{2}$ = 0.42). MGA increased as the size of the thumb and index finger contact surface area increased.

#### Total movement time

**Fig 3:**
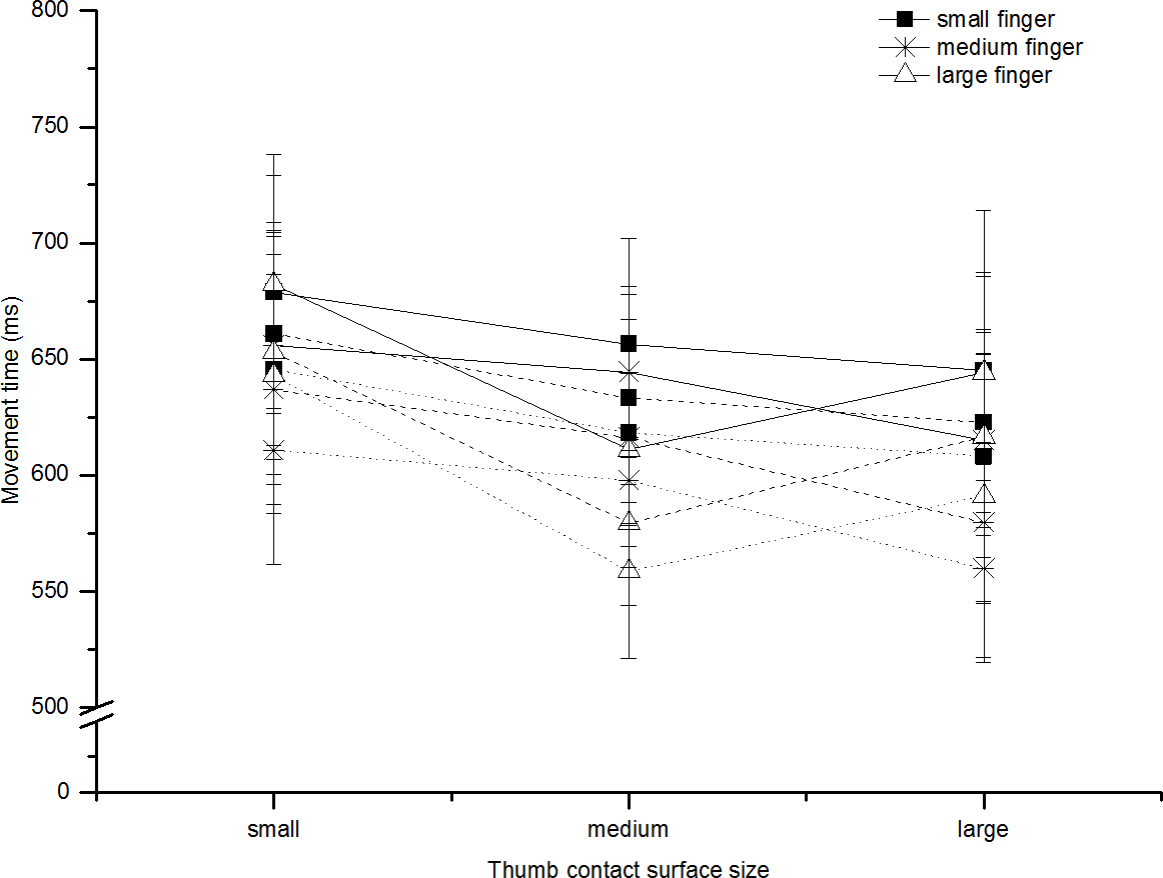
Movement time: Experiment 1b. Shows the average total movement time (ms, solid line), thumb movement time (ms, dashed line), finger movement time (ms, dotted line) for all participants in reaches to all contact surface sizes. The lines represent the finger contact surface (square = small, star = medium, triangle = large). Error bars represent standard error of the mean; these are calculated for within-subjects designs (Cousineau [20] with correction by Morey [21]).

Movement time was not statistically affected by the contact surface for the index finger (F(2,14) = 1.21, p = 0.33, $\eta_{p}^{2}$ = 0.15). However, there was an effect of the contact surface for the thumb (F(2,14) = 7.93, p<0.01, $\eta_{p}^{2}$ = 0.53). Contact surface size influenced the movement of the thumb so that total MT increased as size of the contact surface of the thumb decreased (see Fig 3).

#### Finger and thumb movement time

We additionally studied the impact of the contact surfaces on the movement time of the thumb and index finger (time from onset (>5cm/s) to offset (<5cm/s)). The thumb finished before the finger 33% of the time across all trials and participants and the average difference between the finger and thumb finish was 40ms. The finger’s duration was not affected by the finger contact surface size (F(2,14) = 1.75, p = 0.21, $\eta_{p}^{2}$ = 0.20) but was affected by the thumb contact surface size (F(2,14) = 8.57, p<0.01, $\eta_{p}^{2}$ = 0.55), with duration increasing as surface size decreased. The thumb’s duration was affected by the thumb contact surface size (F(2,14) = 7.04, p<0.01, $\eta_{p}^{2}$ = 0.50) but not the finger contact surface size (F(2,14) = 1.76, p = 0.21, $\eta_{p}^{2}$ = 0.20) again with duration increasing as contact surface size decreased.

### Discussion

The results of Experiment 1a and 1b showed that the size of the contact surface of the thumb played a greater role than that of the finger in determining the total duration of the movements. A larger contact surface for the thumb resulted in shorter duration movements. The size of the effect for the finger surface size was around 20% (Experiment 1a) and 28% (Experiment 1b) of the effect size for the thumb. The Maximum Grip Aperture was influenced by the contact surface size for both digits. We next investigated whether these findings would generalise to other objects and to a different form of prehensile behaviour.

## Experiment 2

Experiment 2 studied whether the findings of Experiment 1a and 1b would generalise to objects grasped in a different plane. We therefore mounted conical frusta (the part of a conical solid left after cutting off the pointed end, see Fig 1) directly in front of the participants (in the sagittal plane along their midline) and asked the participants to grasp the objects with their thumb at the top and their index finger underneath (see Fig 1)–the objects could not be moved so the task had no lifting or rotating component.

### Method

#### Participants

Eight unpaid participants (undergraduate students) were recruited for the study (3 male, 5 female). Participants were aged between 20 and 29 years of age (mean = 24.8 years). All the participants were right handed, all had normal or corrected to normal vision, and none had any history of neurological deficit. Participants provided their written informed consent prior to their inclusion in the study. The study was approved by the School of Psychology, University of Aberdeen ethics committee, and was performed in accordance with the ethical standards laid down in the Declaration of Helsinki.

#### Experimental set-up and procedure

The experimental task required participants to sit at a table and reach-to-grasp (between their index finger and thumb) conical frusta located along the participant’s midline (sagittal plane) at a height of 15cm above the table top. Nine objects were used: The length of all objects was 5 cm, but the size of the grasping surfaces to be contacted by the thumb and index finger varied (diameter 1, 2 or 3 cm). Three of the objects were cylinders as they had the same contact surface size for the thumb and the index finger.

The starting position was defined by a small pea-sized moulded grip located along the participant’s midline, which was held by the participant between the thumb and index finger. The rest of the digits were folded into the palm, and the hand rested on the table top in a comfortable neutral posture. The reach-to-grasp movements were performed with the preferred hand. The object to be grasped was placed at one of three horizontal distances from the starting point (10cm, 30cm or 50cm). Each participant performed 10 trials at each distance and contact surface size combination, resulting in a total of 270 trials. Data acquisition and analysis was identical to that reported in Experiment 1b, and all dependent variables were calculated in the same way.

#### Design

The experiment was a within-subjects design. All participants performed reaches to each combination of contact surface size at each distance. The order of size/distance combinations was randomised across and within the participants; however, participants performed 10 trials in succession at each configuration. The independent variables manipulated were the distance of the target and the contact surface size to be contacted by the finger and thumb. The dependent variables of interest were maximum grip aperture (MGA), total movement time, and movement time of the thumb and finger. The median value of the ten trials performed to each target object were then entered into a repeated measures ANOVA, using thumb (3 contact surface sizes: 1, 2 and 3 cm), finger (3 contact surface sizes: 1, 2, and 3 cm) and distance (10, 30 and 50cm) as within-subjects variables. Again, means were calculated from median values and all means not presented in Figures can be found in Table 1.

### Results

#### Maximum grip aperture (MGA)

MGA was influenced by the contact surface for the thumb (F(2,14) = 23.01, p<0.001, $\eta_{p}^{2}$ = 0.77) and index finger (F(2,14) = 32.15, p<0.001, $\eta_{p}^{2}$ = 0.82). MGA increased as the size of the thumb and index finger contact surface area increased. MGA was not significantly affected by distance (F(2,14) = 3.67, p = 0.06, $\eta_{p}^{2}$ = 0.34).

#### Total movement time

**Fig 4:**
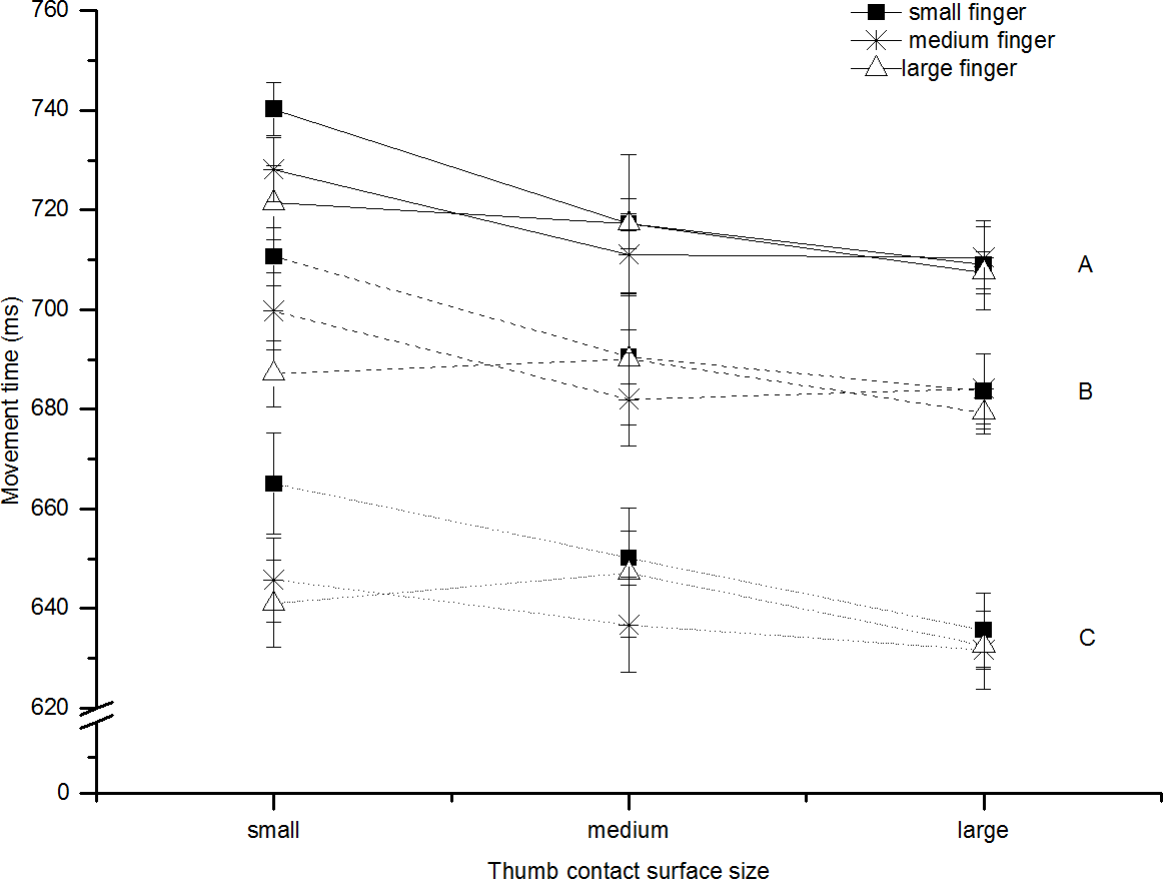
Movement time: Experiment 2. Shows A) the average total movement time (ms, solid line), B) thumb movement time (ms, dashed line), C) finger movement time (ms, dotted line) for all participants in reaches to all contact surface sizes (collapsed across distance). The lines represent the finger contact surface size (square = small, star = medium, triangle = large). Error bars represent standard error of the mean; these are calculated for within-subjects designs (Cousineau [23] with correction by Morey [24]).

Movement time increased as a function of target distance (F(2,14) = 271.27, p<0.001, $\eta_{p}^{2}$ = 0.98). MT was not statistically affected by the contact surface for the index finger (F(2,14) = 0.54, p = 0.60, $\eta_{p}^{2}$ = 0.07). However, there was an effect of the contact surface for the thumb (F(2,14) = 8.03, p<0.01, $\eta_{p}^{2}$ = 0.53). Contact surface size influenced the movement of the thumb so that total MT increased as size of the contact surface of the thumb decreased (see Fig 4).

#### Finger and thumb movement time

We additionally studied the impact of the contact surfaces on the time at which the thumb and index finger first fell below the 5cm/s threshold. The thumb finished before the finger 18% of the time across all trials and participants and the average difference between the finger and thumb finish was 65ms. The duration of the thumb’s movement increased as target distance increased (F(2,14) = 252.91, p<0.001, $\eta_{p}^{2}$ = 0.97) and this was also true for the index finger (F(2,14) = 325.36, p<0.001, $\eta_{p}^{2}$ = 0.98). The thumb’s duration was not affected by the finger contact surface size (F(2,14) = 1.07, p = 0.37, $\eta_{p}^{2}$ = 0.13) but was affected by the thumb contact surface size (F(2,14) = 3.77, p = 0.049, $\eta_{p}^{2}$ = 0.35) with duration increasing as surface size decreased. The index finger’s duration was not reliably affected by the thumb contact surface size (F(2,14) = 2.6, p = 0.11, $\eta_{p}^{2}$ = 0.27) or the finger contact surface size, but the latter approached significance (F(2,14) = 3.20, p = 0.07, $\eta_{p}^{2}$ = 0.31).

### Discussion

The results of Experiment 2 extended the findings of Experiment 1 by replicating the general pattern of results using different objects requiring a qualitatively different grasping action. Once again, the pattern of the movement was affected by both contact surfaces as the hand approached the target (so that maximum grip aperture was affected by the thumb and finger contact surface size). Nevertheless, the total movement duration was driven by the size of the contact surface of the thumb in both experiments. It is notable that the thumb contact surface was visible in both Experiment 1 and 2 whereas the contact surface of the index finger was occluded from view. We therefore decided to manipulate the effect of seeing the contact surface.

## Experiment 3

To investigate the influence of the finger contact surface being visible rather than the thumb contact surface, Experiment 3 involved participants grasping the conical frusta so that their index finger was at the top of the object. All other aspects of the design were identical to Experiment 2. Again, all means not presented in Figures can be found in Table 1.

### Method

#### Participants

Eight unpaid participants (undergraduate students) were recruited for the study (4 male, 4 female). Participants were aged between 20 and 22 years of age (mean = 21 years). All the participants were right handed, all had normal or corrected to normal vision, and none had any history of neurological deficit. Participants provided their written informed consent prior to their inclusion in the study. The study was approved by the School of Psychology, University of Aberdeen ethics committee and was performed in accordance with the ethical standards laid down in the Declaration of Helsinki.

### Results

#### Maximum grip aperture (MGA)

MGA was influenced by the contact surface for the index finger (F(2,14) = 12.90, p<0.001, $\eta_{p}^{2}$ = 0.65) and thumb (F(2,14) = 11.84, p<0.001, $\eta_{p}^{2}$ = 0.63). MGA increased as the size of the thumb and finger contact surface area increased. MGA was not significantly affected by distance (F(2,14) = 0.31, p = 0.74, $\eta_{p}^{2}$ = 0.04).

#### Total movement time

**Fig 5:**
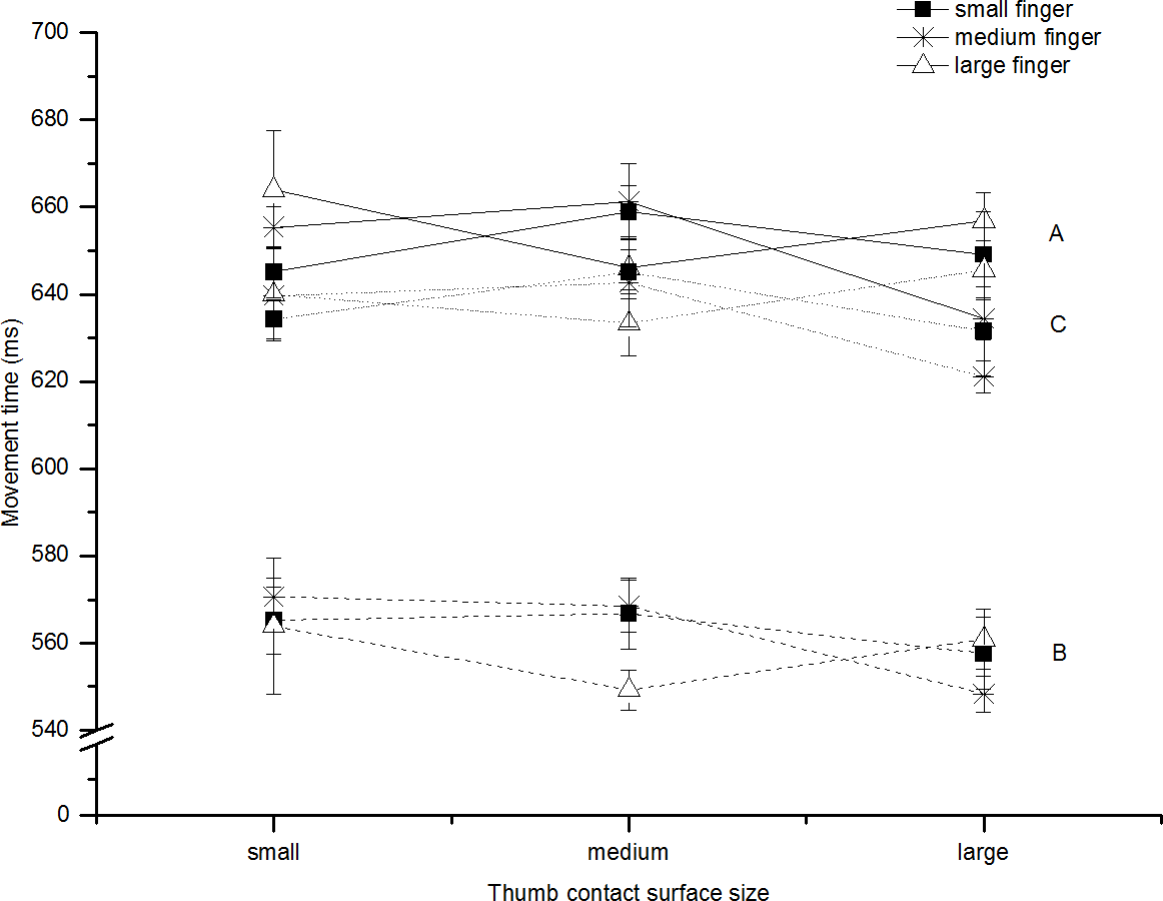
Movement time: Experiment 3. Shows A) the average total movement time (ms, solid line), B) thumb movement time (ms, dashed line) C) finger movement time (ms, dotted line) for all participants in reaches to all contact surface sizes (collapsed across distance). The lines represent the finger contact surface (square = small, star = medium, triangle = large). Error bars represent standard error of the mean; these are calculated for within-subjects designs (Cousineau [23] with correction by Morey [24]).

Total movement time increased as target distance increased (F(2,14) = 102.21, p<0.001, $\eta_{p}^{2}$ = 0.94). There were no main effects for the contact surface for the index finger (F(2,14) = 0.40, p = 0.68, $\eta_{p}^{2}$ = 0.06) or thumb (F(2,14) = 0.70, p = 0.51, $\eta_{p}^{2}$ = 0.09), but there was a significant interaction between the effect of the contact surfaces for the thumb and the index finger (F(4,28) = 3.55, p = 0.02, $\eta_{p}^{2}$ = 0.34). The interaction can be seen in Fig 5.

#### Finger and thumb movement time

We again studied the impact of the contact surface size on the time at which the thumb and index finger first fell below the 5cm/s threshold. The thumb finished before the finger 86% of the time across all trials and participants and the average difference between the finger and thumb finish was 86ms. The duration of the thumb’s movement increased as target distance increased (F(2,14) = 58.01, p<0.001, $\eta_{p}^{2}$ = 0.89) and this was also true for the index finger (F(2,14) = 109.43, p<0.001, $\eta_{p}^{2}$ = 0.94). The thumb’s duration was not affected by the thumb surface (F(2,14) = 0.98, p = 0.40, $\eta_{p}^{2}$ = 0.12) or the finger surface (F(2,14) = 0.25, p = 0.78, $\eta_{p}^{2}$ = 0.03). The index finger’s duration was also not reliably affected by the finger’s surface (F(2,14) = 0.33, p = 0.73, $\eta_{p}^{2}$ = 0.04) or the thumb surface (F(2,14) = 0.49, p = 0.62, $\eta_{p}^{2}$ = 0.07).

### Discussion

The results of Experiment 3 were different to the results of Experiments 1 and 2. There was now an interaction between the thumb’s contact surface size and the contact surface size for the index finger, so the surface size of both digits influenced total movement time. Notably, a larger contact surface size caused an increased movement duration in some conditions in Experiment 3 (whereas a larger contact surface size was associated with decreased duration in Experiments 1 and 2). This result can be explained by the object’s increased surface area creating a larger obstacle to the approaching digit (with prehension necessarily entailing obstacle avoidance as well as digit placement). Notably, the larger contact surfaces caused an increased MGA across all three experiments. The increased MGA makes sense with larger surfaces as it provides a greater safety margin for avoiding collision as the digits close on the object. These results are therefore consistent with the conclusion that larger surfaces increase the possibility of collision but decrease the accuracy demands of the task. In the first two experiments, the decreased accuracy demands had the largest effect (causing a lower movement duration) but in Experiment 3 (where the thumb was no longer visible), it appeared to be the increased possibility of collision that affected the duration (causing a longer duration in some conditions).

## General discussion

The results of the series of experiments provide the first empirical data that address the issue of the duration of prehensile movements to objects that have asymmetrical contact surfaces. The results of Experiments 1 (a and b) and 2 indicate that the total task duration was primarily a function of the thumb’s contact surface, with the index finger contact surface not having a statistically significant effect on total movement time. In Experiments 1 and 2, the contact surface for the thumb was visible whilst the contact surface for the index finger was occluded from view. In Experiment 3, the contact surface for the index finger was visible whilst the thumb’s surface was occluded. It might be expected that this arrangement would change the findings of Experiment 1 and 2, and indeed we observed an interaction between the effect of the thumb and index finger surface size on total duration.

Experiment 3 is consistent with the results of Melmoth & Grant [25] who found that visual feedback was used to guide the thumb in the period just prior to contacting the object, with the finger being involved in avoiding collision with the opposite contact surface. This makes good sense when one considers that the anatomy of the arm and hand means that the thumb is more frequently the digit that can be visually monitored as it contacts the surface of an object (in both right and left handers). Therefore, while it is reasonable to expect that particular task constraints can cause the index finger to be the transported endpoint, there is a natural bias to transport the thumb. This observation can explain why there are mixed results within the literature regarding the evidence for whether the thumb or index finger (or both) are transported in reach-to-grasp movements [13–18, 26].

The vector account of prehension provided by Mon-Williams and Bingham [3] assumes that the thumb is the preferred origin of the grasp vector as it has evolved in humans to have a large fleshy pad that offers a stable base for precision grips [12]. Moreover, human anatomy means that it is typically the thumb surface that is visible when reaching-to-grasp objects. Mon-Williams and Bingham [3] suggested, however, that there is flexibility in the digit selected as the origin of the grasp vector. They specifically suggested that the point of fixation would influence the origin selection. The present experiments appear to provide support for this conjecture. In Experiment 1 and 2 the thumb’s contact surface size alone determined overall duration, and this was the visible surface. The pattern was less clear in Experiment 3, however, where both contact surfaces influenced the total movement duration despite only the index finger surface being visible. This supports the vector account’s assumption that there is a bias towards the thumb acting as the origin (i.e. the thumb serving as the transported endpoint), but the index finger can also act in this role when visual constraints make this advantageous.

The general idea that the fixation point plays an important role in the determination of the prehensile movement patterns has support from a study conducted by Ross, Schenk and Hesse [27]. Ross, Schenk and Hesse found that obstacle avoidance behaviour was moderated by fixation position when participants were required to move their hands through a gap between two obstacles into a target area. There is also support for this idea from experiments conducted by Desanghere and Marotta [28] and Voudouris, Smeets and Brenner [26]. In an apparent change from an earlier conclusion that humans do not aim for visible grasp points [29], the results of Voudouris, Smeets and Brenner [26] suggest (in agreement with other previous work [22–24]) that it is the index finger that is normally fixated. The findings of Voudouris, Smeets and Brenner [26] indicated that the fixation point is highly influenced by the digit that has the greatest accuracy demands. They also found that participants minimised the time during which the eyes are moving and this results in a bias to fixate where they were looking previously. The general bias reported by Voudouris, Smeets and Brenner [26] was to fixate the index finger rather than the thumb. Desanghere and Marotta [28] investigated fixation bias as a function of the centre of mass of the objects being grasped. Desanghere and Marotta found that changes in the centre of mass altered the fixation point, and reported that fixations to asymmetric objects were not as tightly linked to index finger grasp locations as previously reported with symmetrical objects. The overall conclusion that can be drawn from these studies is that task constraints have a large influence on the fixation point in reach-to-grasp movements, with fixation behaviour being subject to various biases. The important point is that the fixation behaviour appears to influence the prehension movement patterns. We are unable to speculate on where our participants were fixating (as we did not record eye movements) but our suggestion that the reach-to-grasp behaviour is a function of fixation is consistent with our findings and a number of reports in the literature.

In conclusion, we have established an influence of asymmetric contact surfaces on the total time taken to reach-and-grasp objects. It is our hope that these data will allow scientists to improve their predictions regarding the influence of object design on reach-to-grasp movements. We anticipate that such predictions will assist in the identification and classification of manual impairment, and will help designers create objects that are well suited to individuals who have specific impairments [30].
